# The relationship between retinal nerve fibre layer thickness profiles and CorvisST tonometry measured biomechanical properties in young healthy subjects

**DOI:** 10.1038/s41598-017-00345-y

**Published:** 2017-03-24

**Authors:** Masato Matsuura, Hiroshi Murata, Shunsuke Nakakura, Yoshitaka Nakao, Takehiro Yamashita, Kazunori Hirasawa, Yuri Fujino, Yoshiaki Kiuchi, Ryo Asaoka

**Affiliations:** 1 Department of Ophthalmology, The University of Tokyo, Tokyo, Japan; 2 Department of Ophthalmology, Saneikai Tsukazaki Hospital, 68-1, Waku, Aboshi-ku, Himeji, Hyogo 671-1227 Japan; 3 Department of Ophthalmology and Visual Science, Hiroshima University, 1-3-2 Kagamiyama Higashihiroshima, Hiroshima, 739-8511 Japan; 4 Department of Ophthalmology Kagoshima University Graduate School of Medical and Dental Sciences, Kagoshima, Japan; 5 Department of Orthoptics and Visual Science, School of Allied Health Sciences, Kitasato University, 1-15-1 Kitasato, Minami-ku, Sagamihara, Kanagawa 252-0373 Japan

## Abstract

We previously reported that a shallow circumpapillary retinal nerve fiber layer (cpRNFL) peak angle as measured by optical coherence tomography (OCT) suggests the temporal retina is stretched around the optic disc from the papillo-macular bundle (Yamashita T *et al.* Investigative Ophthalmol Vis Sci 2013). The purpose of the current study was to investigate the relationship between CorvisST tonometry (CST) corneal measurements, axial length (AL) and the change in OCT-measured cpRNFL peak angle, in young healthy subjects. OCT and CST measurements were carried out in 97 eyes of 97 young healthy volunteers. The relationship between cpRNFL peak angle and 12 CST parameters, adjusted for AL, was investigated using linear modelling. The mean ± standard deviation cpRNFL peak angle of the 97 healthy volunteers was 130.6 ± 25.4 (range: 77.8 to 207.0) degrees. The optimal linear model to explain cpRNFL peak angle (chosen from 2^16^ different models) included three CST variables related to the speed and size of energy absorption (namely, A1 time, A1 length and A2 time), in addition to AL. In eyes with longer AL and shorter energy absorption in CST measurement, temporal retina is stretched around the optic disc from the papillo-macular bundle, as suggested by a shallow cpRNFL peak angle.

## Introduction

The retinal nerve fiber layer (RNFL) is located in the innermost layer of the retina, and is comprised of the axons of retinal ganglion cells. A decrease in the thickness of this layer is observed in a number of ocular diseases including glaucoma, which is the second commonest cause of blindness worldwide^1, 2^. The development of optical coherence tomography (OCT) has enabled thickness measurements of the RNFL^3–6^ however, the OCT-measured thickness profile around the optic disc is influenced by the elongation of the eye ball and axial length^7–9^. There are two peaks in the circumpapillary RNFL (cpRNFL) thickness profile: the supratemporal and infratemporal peaks. We have previously analyzed the relationship between these peaks and axial length (AL) in young healthy subjects. This research suggested that the temporal retina is stretched around the optic disc from the papillo-macular bundle in eyes with a shallow cpRNFL peak angle^9^. However, the association between AL and peak angle was relatively weak (correlation coefficient = −0.49), so other factors must also influence the change in cpRNFL profile. One known example is body height^10^, but other ocular factors may also play an important role.

**Figure 1:**
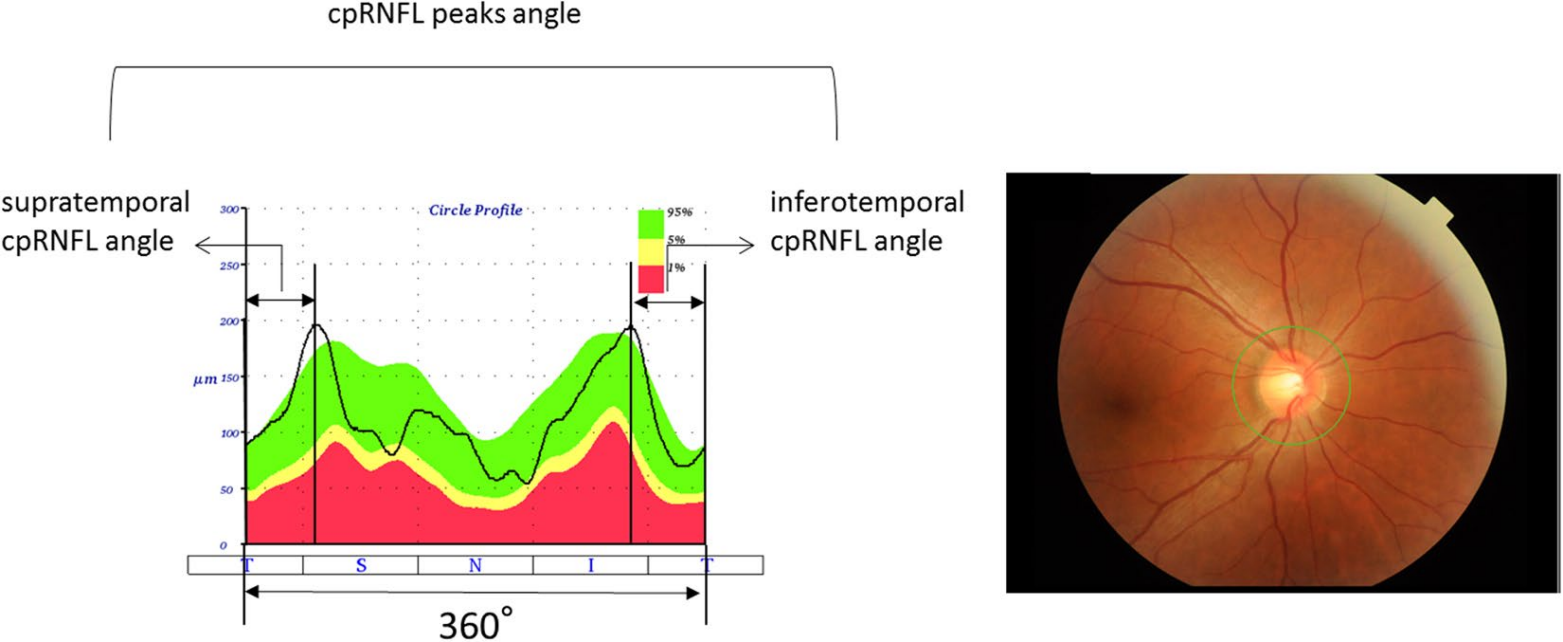
Calculation of cpRNFL peaks angle. The cpRNFL peaks angle was calculated as the angle between the supratemporal cpRNFL peak angle and inferotemporal cpRFNL peak angle. cpRNFL: circumpapillary retinal nerve fiber layer.

The rigidity of the eye may also be correlated to the measured cpRNFL profile, being related with the elongation of axial length. Corneal hysteresis is a biomechanical measure of the cornea’s ability to absorb and dissipate energy. ‘Hysteresis’ is a physical term describing a property of a material that does not instantly react to an applied force, and it can be thought of as the amount of energy absorption during the ‘loading/unloading’ stress/strain cycle^11, 12^. The Corneal Visualization Scheimpflug Technology instrument (CorvisST tonometry: CST; Oculus, Wetzlar, Germany) measures the cornea’s viscoelastic properties by imaging the response of the cornea to pressure by an air jet. In CST, detailed corneal movements are examined using the integrated ultra-high-speed Scheimpflug camera^13^, such as the time and the width of corneal deformation following an air puff application (Fig. 1). CST measures biomechanical properties of the cornea and we have recently reported that CST parameters are significantly related to corneal hysteresis measured with the Ocular Response Analyzer (ORA, Reichert Ophthalmic Instruments, Depew, NY, USA), which measures air jet pressure at first and second applanation events^14^.

It is of interest to investigate whether CST parameters are associated with the measured cpRNFL profile, after adjustment for axial length. This may help us to understand the relationship between myopia and retinal structural changes. This is particularly important in areas where the prevalence of myopia is high. For instance, in Japan, the Tajimi study reported that the incidence of myopia in the Japanese population was 41.8% (defined as >−0.5 diopters: D) and 5.5% for high myopia (>−6.0 D) in individuals aged 40 years and older^15^. The prevalence of high myopia is also elevated in other Asian countries^16, 17^. Also, high myopia is a strong risk factor for glaucoma^18, 19^, and, further, a recent study suggested that maximum deformation amplitude – measured by CST – is associated with the size of *β*-zone parapapillary atrophy^20^ which is linked to the development, severity, progression and location of glaucoma^21–24^. Curiously, in a different study no relationship was observed between ORA-measured corneal hysteresis and *β*-zone parapapillary atrophy^25^. Research also suggests that maximum deformation amplitude is associated to optic disc tilt, which, in turn, is closely related to myopia^26, 27^.

Understanding the effects of axial length, myopia and other ocular biomechanics on retinal measurements may be important to understand the development and progression of glaucoma. In the current study, CST and OCT measurements were performed in young healthy volunteers, and the association of CST measured corneal parameters and the OCT-measured cpRNFL profile was investigated. As a result, it was shown that low hysteresis of cornea measured with CST, in addition to elongated axial length, is significantly related to stretched temporal retina around optic disc from papillo-macular bundle, as suggested by shallow cpRNFL peaks angle.

## Methods

The study protocol was approved by the institutional review board of university of Tokyo, University of Hiroshima, and Tsukazaki Hospital and adhered to the tenets of the Declaration of Helsinki. Written informed consent was obtained from each subject.

### Subjects

This was a cross sectional, prospective, observational study. We studied 97 eyes of 97 young healthy volunteers who were enrolled between February 2016 and August 2016 at either of university of Tokyo, University of Hiroshima, and Tsukazaki Hospital. The participants had no known eye diseases as determined by examining their medical history. Data from one randomly chosen eye was included in the study. To mitigate aging effects, only young adults were recruited to this study. Therefore, the inclusion criteria were: aged between 20 and 40 years; no pathological findings by slit–lamp biomicroscopy, ophthalmoscopy, or OCT; best corrected visual acuity (BCVA) ≧0.1 logarithm of the minimum angle of resolution (logMAR) units. Exclusion criteria were: known ocular diseases such as glaucoma, staphyloma, and optic disc anomalies; systemic diseases such as hypertension and diabetes; presence of VF defects; and history of refractive or intraocular surgery. None of the eyes was excluded because of poor OCT image quality caused by poor fixation. Intraocular pressure (IOP) was measured with Goldmann applanation tonometry (GAT) three times and the average value was used in the analysis. All eyes had an average IOP value of ≤21 mmHg.

### Measurement of axial length, central corneal thickness and body height

Axial length (AL) was measured with the IOL master (Carl Zeiss Meditec, Dublin, CA, USA). Central corneal thickness (CCT) was measured with CST three times and the average value was used in the analysis. Just prior to the ocular examination, body height was measured with participants standing without shoes.

### Angles between supratemporal and infratemporal RNFL thickness peaks (cpRNFL peak angle)

RNFL thickness was measured with the TOPCON 3D OCT-2000 MARK II using the RNFL 3.4 mm circle scan, 1024 A-scans/circle, 16 overlapping B-scans/image, and direct B-scan observations. In the OCT measurement, a color fundus photograph was recorded at the same time as the OCT measurement. The edge of the optic disc in the fundus image was automatically specified by the optical system of the OCT instrument and the scan circle was centered on the defined center of the optic disc. In the analysis, the center of the scan circle was used as the center of the disc. The coordinates of each pixel were determined automatically using the ImageJ program (Image J version 1.47, National Institutes of Health, Bethesda, MD, USA; http://imagej.nih.gov/ij/ [in the public domain]). Then the temporal-superior-nasal-inferior-temporal (TSNIT) cpRNFL thickness curve was profiled to measure the angle between the supra-temporal and infra-temporal peaks of the cpRNFL thickness profile. Then, the distance between supra-temporal or infra-temporal RNFL peaks was converted to an angular value by dividing it by the entire distance, and multiplying by 360^28^. A case example to calculate the cpRNFL peak angle is shown in the Fig. 1.

### Corvis ST tonometry

**Figure 2:**
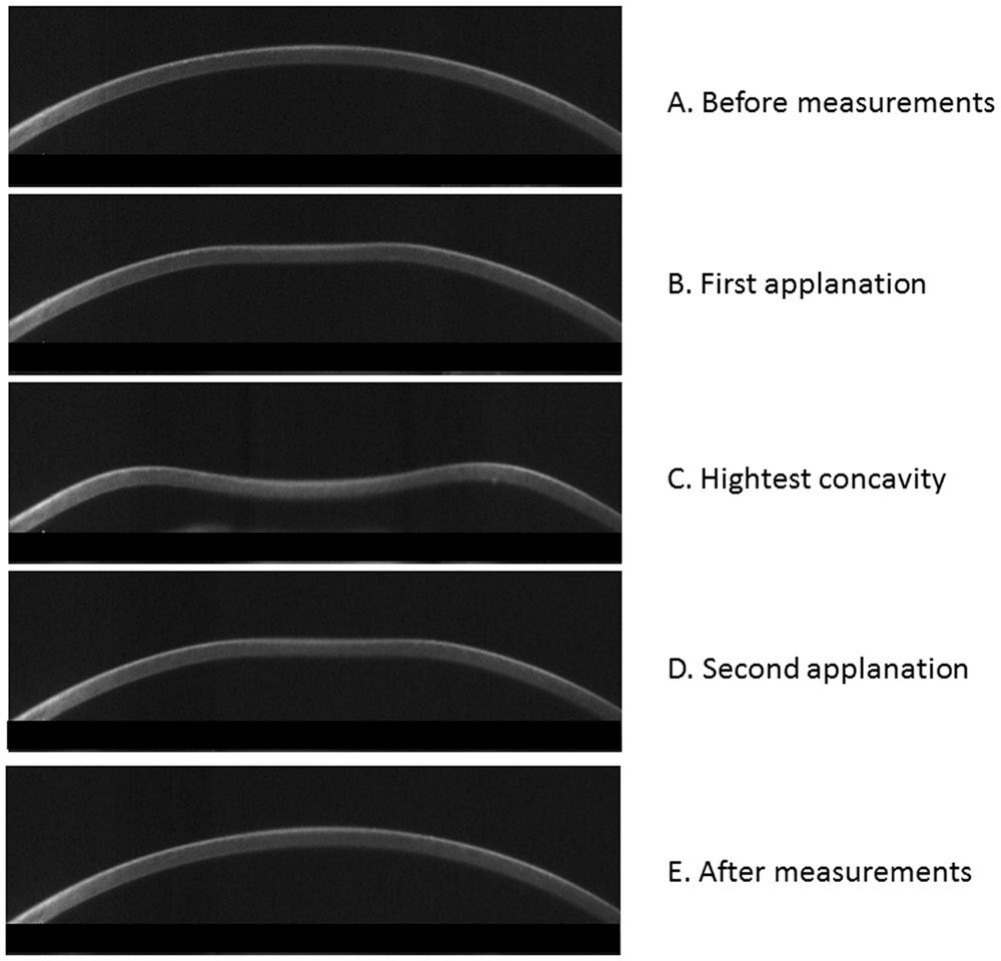
Corneal movement during the CST measurement. In the CST tonometry measurement, a rapid air puff is applied to cornea and cornea moves inward. The figures show the corneal shape in each phase: (**A**) Prior to air puff applanation, (**B**) first applanation, (**C**) highest concavity, (**D**) second applanation, and (**E**) posterior to air puff applanation. CST: Corvis ST tonometry.

The measurement principles of CST are described elsewhere^13^. In short, the instrument’s camera records a sequence of images (capturing up to 4,330 images per second) that capture corneal deformation by the application of a rapid air puff (Fig. 2). A total of 12 CST parameters are captured (see Table 1): ‘A1 and A2 time’ is the duration of time from the initiation of the air puff to the first (inward) or second (outward) applanations, respectively; ‘A1 and A2 length’ is the length of the flattened surface of cornea at the first or second applanation, respectively; ‘A1 and A2 velocity’ is the velocity of the cornea apex movement during the first or second applanation, respectively; ‘A1 and A2 deformation amplitude’ is the magnitude of the corneal apex movement at the first or second applanation, respectively; ‘peak distance’ is the distance between the two surrounding peaks of the cornea at the highest concavity; ‘highest deformation amplitude’ is the magnitude of movement of the corneal apex from before deformation to the maximum concavity, ‘highest concavity time’ is the duration taken to reach maximum concavity from pre-deformation of the cornea; and ‘radius’ is the central curvature radius at the maximum concavity.

**Table 1:** Measured CST parameters.

CST Parameters	Meaning	Mean ± SD	Range
A1 time (ms)	length of time from the initiation of the air puff to the first applanation	7.3 ± 0.2	6.9 to 8.0
A1 length (mm)	length of the flattened corneal surface at the first applanation	1.8 ± 0.04	1.7 to 1.9
A1 velocity (m/s)	velocity of the movement of cornea apex during the first applanation	0.16 ± 0.01	0.12 to 0.18
A1 deformation amplitude (mm)	magnitude of the movement of the corneal apex at the first applanation	0.12 ± 0.01	0.09 to 0.15
A2 time (ms)	length of time from the initiation of the air puff to the second applanation	21.8 ± 0.4	21.0 to 22.7
A2 length (mm)	length of the flattened corneal surface at the second applanation	1.7 ± 0.2	1.0 to 2.2
A2 velocity (m/s)	velocity of the movement of cornea apex during the second applanation	−0.40 ± 0.06	−0.56 to −0.25
A2 deformation amplitude (mm)	magnitude of the movement of the corneal apex at the second applanation	0.35 ± 0.06	0.24 to 0.53
highest deformation amplitude (mm)	magnitude of movement of the corneal apex from before deformation to the highest concavity	1.1 ± 0.09	0.87 to 1.3
highest concavity time (ms)	duration taken to reach highest concavity from pre-deformation of the cornea	16.6 ± 0.4	14.8 to 17.5
Peak distance (mm)	distance between the two surrounding peaks of the cornea at the highest concavity	4.6 ± 0.9	2.3 to 5.5
Radius (mm)	central curvature radius at the highest concavity	7.0 ± 0.8	5.4 to 9.0

CST: Corvis ST tonometry, SD: standard deviation.

The CST measurement was carried out three times prior to the GAT-IOP measurement and the averages of CST parameters were calculated. CST measurements were considered reliable according to the “OK” quality index displayed on the CST device monitor. All of the measurements were performed on the same day.

### Statistical analyses

The relationship between cpRNFL peak angle and age, CCT, AL and body height was investigated using a multivariate regression analysis. In addition, an optimal linear model was selected among all possible combinations (2^16^) of the following predictors: age, CCT, AL, body height and 12 CST parameters. The second order bias corrected Akaike Information Criterion (AICc) index was used to determine the optimal model^27^. As introduced in our previous paper^29^, any magnitude of reduction in AICc suggests an improvement of the model fit, and the probability that a particular model minimizes ‘information loss’ can be calculated as follows^30^; when there are *n* candidate models and the AICc values of those models are AIC*1*, AIC*2*, AIC*3*, …, AIC*n*. If AIC*min* is the minimum of these values then exp((AIC*min* − AIC*i*)/2) describes the relative probability that the *i*th model minimizes the information loss (i.e. it is the ‘optimal model’).

The relationship between cpRNFL peak angle and the other parameters was also investigated using Pearson’s correlation. All statistical analyses were performed with the statistical programming language ‘R’ (R version 3.1.3; The Foundation for Statistical Computing, Vienna, Austria).

## Results

**Figure 3:**
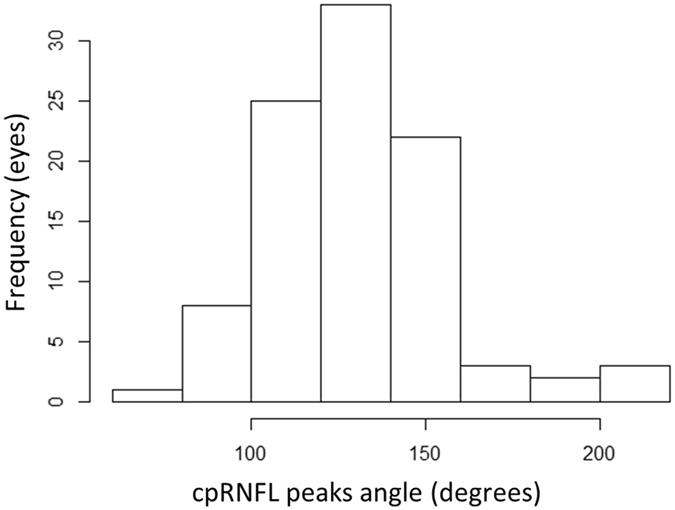
Histogram of cpRNFL peaks angle. The cpRNFL peaks angle was 130.6 ± 25.4 (mean ± standard deviation) [range: 77.8 to 207.0] degrees. cpRNFL: circumpapillary retinal nerve fiber layer.

Table 1 summarizes the measured CST parameter values. Characteristics of the study population are summarized in Table 2. Subjects’ average age was 27.7 ± 5.0 (mean ± standard deviation) [range: 21–39] years. The mean axial length was 25.2 ± 1.3 [22.6–28.5] mm. The mean cpRNFL peak angle was 130.6 ± 25.4 (77.8 to 207.0) degrees (Fig. 3). The univariate relationship between cpRNFL peak angle and age, body height, CCT, GAT IOP, axial length and the 12 CST parameters is summarized in Table 3; AL, A1 deformation amplitude and A2 time were significantly correlated with cpRNFL peak angle (p < 0.05).

**Table 2:** Subject demographics and summary ocular measurements.

Variables	Value
age, (mean ± SD) [range], years old	27.7 ± 5.0 [21 to 39]
Male/Female	47/50
Right/Left	89/8
GAT IOP, (mean ± SD) [range], mmHg	14.3 ± 3.0 [7.3 to 20.7]
AL, (mean ± SD) [range], mm	25.2 ± 1.3 [22.6 to 28.5]
CCT, (mean ± SD) [range], μm	529.0 ± 34.5 [458.3 to 624.3]
cpRNFL peaks angle, (mean ± SD) [range], degrees	130.6 ± 25.4 [77.8 to 207.0]

GAT: Goldmann applanation tonometry, IOP: intraocular pressure, AL: axial length, CCT: central corneal thickness, cpRNFL: circumpapillary retinal nerve fiber layer, CST: Corvis ST tonometry, SD: standard deviation.

**Table 3:** Correlation between cpRNFL peak angle and ocular parameters.

CST Parameters	Correlation coefficient	p value
Age	0.15	0.15
CCT	0.006	0.95
body height	−0.18	0.082
AL	−0.27	0.009
A1 time (ms)	−0.057	0.58
A1 length (mm)	0.098	0.34
A1 velocity (m/s)	−0.00071	0.99
A1 deformation amplitude (mm)	−0.22	0.031
A2 time (ms)	0.29	0.0038
A2 length (mm)	0.099	0.33
A2 velocity (m/s)	0.037	0.72
A2 deformation amplitude (mm)	0.18	0.08
highest deformation amplitude (mm)	0.051	0.62
highest concavity time (ms)	0.14	0.17
Peak distance (mm)	0.093	0.37
Radius (mm)	−0.12	0.23

cpRNFL: circumpapillary retinal nerve fiber layer, CST: Corvis ST tonometry, CCT: central corneal thickness, AL: axial length.

As shown in Table 4, the adjusted R^2^ value and the AICc value associated with the multivariate regression model between cpRNFL peak angle against age, body height, CCT and axial length was 0.062 and 904.2, respectively. In this model, only axial length (coefficient = −4.3, p = 0.030) was a significant predictor of cpRNFL peak angle. The optimum model for cpRNFL peak angle chosen from 2^16^ models was: cpRNFL peaks angle = −901.1 + 132.7 × A1 length + 27.8 × A1 time + 31.0 × A2 time – 3.4 × axial length. The adjusted R^2^ value and AICc values of this model were 0.15 (p = 0.00089) and 895.1, respectively (see Table 4). The relative probability that this was the optimum model compared with the model with only four variables (age, body height, CCT and axial length; R^2^ value = 0.062, p = 0.042) was 99.0%.

**Table 4:** Linear model results to explain cpRNFL peak angle.

	Coefficient	Standard error	p value
**Multiple regression**			
age	0.72	0.50	0.15
CCT	−0.011	0.081	0.89
body height	−0.31	0.31	0.32
AL	−4.3	2.0	0.030
adjusted R^2^	0.062 (p = 0.042)		
AICc	904.2		
**Optimum model**			
A1 length	132.7	62.9	0.038
A1 time	27.8	13.8	0.047
A2 time	31.0	9.3	0.0012
AL	−3.4	1.9	0.073
adjusted R^2^	0.15 (p = 0.00089)		
AICc	895.1		

cpRNFL: circumpapillary retinal nerve fiber layer, CCT: central corneal thickness, AL: axial length, AICc: corrected Akaike information criteria.

## Discussion

In this study the relationship between cpRNFL peak angle and axial length and 12 CST parameters was investigated. The optimal model to explain cpRNFL peak angle included a number of CST parameters, namely A1 length, A1 time, A2 time and also AL. A shallower cpRNFL peak angle was associated with shorter A1 length, shorter A1 time, shorter A2 time and longer AL. In agreement with previous studies^7, 9^ cpRNFL angle was more shallow in eyes with longer AL. A fundus photograph of a case example showing RNFL bundles shifted towards the papillo-macular bundle in a myopic eye is given in our previous report^9^. This finding is not only important to understand retinal structure in eyes with myopia, but also to understand the development of structural changes in eyes with glaucoma. In myopic early-stage glaucomatous eyes, RNFL defects often appear at the paracentral area, and we have shown that RNFL defects locate closer to the papillo-macular bundle as AL increases^31^. This finding has also been reported in an independent study^32^. Further, in myopic glaucomatous eyes, cecocentral scotomas located slightly temporal and inferior to the fixation point are observed with high frequency^33, 34^. A shallow cpRNFL peak angle in eyes with a long AL, as suggested in the current result, may explain the spatial preferences of scotomas in myopic eyes. Furthermore, an elongated AL is significantly associated with supernormal RNFL measurements and false-positive sectors in healthy eyes^35^. Thus, careful consideration is needed when interpreting cpRNFL thickness profiles to diagnose glaucoma in eyes with increased AL.

Jung *et al.* reported that eyes with a deep highest deformation amplitude have a large *β* parapapillary atrophy^20^. However, we did not find that highest deformation amplitude was significantly associated with cpRNFL peak angle in the univariate analysis (see Table 3) or in the optimum linear model (see Table 4). In the optimum model to expalin cpRNFL peak angle, the variables of A1 length, A1 time, A2 time and AL were selected; cpRNFL peak angle was shallow in eyes with shorter A1 length, shorter A1 time and shorter A2 time, in addition to longer AL. Hysteresis is a term to describe a property of a physical system that does not instantly follow an applied force but that reacts slowly and it can be thought of as the amount of energy absorption during the ‘loading/unloading’ stress/strain cycle^11, 12^. When an air puff pressure is applied to an eye and the A1 length, A1 time and A2 time are short, the applied energy is poorly absorbed by the cornea; because the absorption phase of the applied energy (the air puff pressure) occurs in a narrow area and in a short period^14^. The posterior pole of the retina is stretched in a posterior (axial) direction by the elongation of eye ball; the shape of the posterior pole is sharper in more elongated eyes, and, as a result, the measured cpRNFL peak angle is likely to be shallower^5, 10^. Thus the current study suggested, in eyes where CST-measured A1 length, A1 time and A2 time are short (corneal energy absorption is poor), the posterior pole of the retina is more sharply stretched, beyond what is attributable to the effect due to an elongation of the eye ball. Clearly, AL can only explain some of the variation in cpRNFL peak angle, as suggested by the significant relationship between AL and cpRNFL peak angle, however the adjusted R^2^ was very small (0.062). There are large variations in AL at birth^36^, and in adult AL, and hence, two adult eyes with an identical AL are unlikely to have shared the same AL at birth. Merely considering AL to describe the magnitude of retinal stretch is not sufficient and instead it is more appropriate to concurrently investigate the cpRNFL peak angle. Considerable increase in the adjusted R^2^ was observed by including CST parameters (0.15), however it should be noted that the adjusted R^2^ was still small even with our optimal linear model using CST parameters to explain cpRNFL peak angle.

A limitation of the current study is that the participants consisted of young healthy volunteers only. Further investigations should be carried out in eyes with glaucoma. However, OCT-measured cpRNFL peak angle will be effected by glaucomatous damage to the RNFL and instead, positions of retinal arteries could be used as we have previously suggested^9, 29^. A similar or even more obvious tendency could be observed in eyes with glaucoma.

In conclusion, eyes with long ALs have shallower cpRNFL peak angles. The cpRNFL angle is even shallower in eyes with short A1 length, short A1 time and short A2 time, suggesting shorter energy absorption is also important.
